# Individual patient-centered target-driven intervention to improve clinical outcomes of diabetes, health literacy, and self-care practices in Nepal: A randomized controlled trial

**DOI:** 10.3389/fendo.2023.1076253

**Published:** 2023-01-19

**Authors:** Shahina Pardhan, Tirthalal Upadhyaya, Lee Smith, Tara Sharma, Sarita Tuladhar, Bhojraj Adhikari, John Kidd, Raju Sapkota

**Affiliations:** ^1^Vision and Eye Research Institute (VERI), School of Medicine, Anglia Ruskin University, Cambridge, United Kingdom; ^2^Department of Medicine, Gandaki Medical College Teaching Hospital, Pokhara, Nepal; ^3^Center for Health Performance and Wellbeing, Anglia Ruskin University, Cambridge, United Kingdom; ^4^Department of Ophthalmology, Gandaki Medical College Teaching Hospital, Pokhara, Nepal; ^5^Medicine, Bharatpur Hospital, Bharatpur, Nepal

**Keywords:** diabetes control, video education training, patient-centered intervention, culturally appropriate, retinopathy screening

## Abstract

**Purpose:**

To examine the effectiveness of a culturally and linguistically appropriate, patient-centered, target-driven lifestyle intervention with video education training in improving clinical outcomes, health literacy, and diabetic self-care practices in newly diagnosed patients in Nepal.

**Methods:**

A total of 110 participants with newly and consequently diagnosed Type 2 were randomly allocated into intervention (mean age = 45 ± 9.7 years) and control (mean age = 47 ± 12.5 years) groups. Intervention group participants were trained on a culturally and linguistically appropriate diabetic video education program and were given a customized dietary and physical activity plan with specific targets to practice at home. Participants’ compliance was monitored weekly *via* telephone calls. Both groups received the usual treatment from their doctor and were followed up after three months. Outcome measures included changes in: i. diabetic health literacy, diet, and physical activity measured using self-reported questionnaires; and ii. blood glucose (glycated hemoglobin, HbA1c), cholesterol, blood pressure, body mass index, and visual acuity. Clinical outcome measures were blinded from randomization and intervention allocation.

**Results:**

After three months, HbA1c decreased to 6.1% from the baseline value of 7.2% in the intervention group compared to 6.6% in the control group from the baseline value of 7.1% (p <0.05). The intervention group had mean total cholesterol and low-density lipoprotein of 174 and 95.5 mg/dL, which were significantly lower than 186 and 107.5 mg/dL in the control group. Daily white rice consumption decreased by 36.5% in the intervention vs. 4% in the control group (p <0.05). After three months, the intervention group participants exercised more than the control group (p <0.05). All intervention group participants self-initiated retinal screening checks since the baseline visit among which 13% showed early diabetic retinopathy signs compared to 0% in the control group. Health literacy improvement in the intervention group was found to be sustained after three months too.

**Conclusions:**

A culturally appropriate, target-driven lifestyle intervention with video education training is effective in improving clinical outcomes, health literacy, and self-care practice in newly diagnosed diabetic patients in Nepal, i.e., at a time period when effective diabetes control is vital to prevent further complications. The training intervention could be rolled out nationwide in order to reduce the risk of diabetic-related complications and improve people’s quality of life and productivity.

## 1 Introduction

Diabetes is among the leading causes of mortality and morbidity in the adult population, globally (1). It is estimated that there are more than 463 million people living with diabetes worldwide, and this number is projected to rise to 643 million by the year 2030 (2). Nearly two-thirds of the world’s diabetic population lives in South Asia (3–5).

Diabetes has emerged as a major public health threat in Nepal. It is the direct cause of more than 10000 deaths annually and is ranked eleventh for causing the greatest number of disability-adjusted life years (6). Nepal has seen a substantial increase in the prevalence of diabetes. In 2021, there were 1.1 million people living with diabetes in Nepal, an increase of 125% from 10 years before in 2011 (2). In 2017, the World Health Organisation (WHO) estimated that the prevalence of diabetes in Nepal will rise by more than three times by the year 2030, which is higher than the estimated 2.5 times rise in its neighboring country India within the same time frame (7). A recent systematic review and metanalysis published in 2020 of 14 studies from Nepal found the pooled prevalence of diabetes to be 8.5% (5), with notable differences between rural (2.5%) and urban (14.6%) regions (8). A population-based study conducted in Kathmandu valley found Diabetic Retinopathy (DR) to be prevalent in approximately 24% of diabetic patients (9). DR is the most common microvascular complication of diabetes and is said to account for approximately 17% of all blindness in Nepal (10).

Various factors have been proposed to explain the increased risk of diabetes, mainly of Type 2, in South Asians (including Nepalese) compared to other ethnic groups such as Caucasians and Hispanics. While it is possible that genetic differences may contribute to excessive abdominal fat, it has been shown that poor health literacy around diabetes control, lower socioeconomic status, rapid urbanization leading to greater physical inactivity (i.e. not meeting physical activity recommendations), poor dietary choices including excessive consumption of white rice, processed food, sweets, and purified fat, and poor planning or target setting around healthy lifestyles are more important risk factors of diabetes in South Asians (11–15)

### 1.1 Knowledge and awareness about diabetes

Improved knowledge and awareness about diabetes is vital in its self-management. Despite this, research suggests that diabetic patients in Nepal can lack adequate knowledge and awareness about diabetes. A hospital-based study conducted in the far western region of Nepal found that 22.5% of diabetic patients lacked adequate knowledge about the role of diet and physical activity (16). A study conducted in Kathmandu valley found that nearly 24% of diabetic patients did not believe that diabetes is a serious disease (17). Data from the western region of Nepal showed that patients at a higher risk of uncontrolled diabetes, and therefore at an increased risk of complications, had low levels of health literacy surrounding diabetes and its complications, and showed poor compliance to medical treatment (18). Also, patients most at risk of diabetic complications were found to not monitor their cholesterol and blood pressure levels regularly (18). Raised cholesterol and blood pressure levels are associated with poor control of diabetes (19–21).

In addition, in remote villages of Nepal, diabetic patients often visit traditional or faith healers rather than medical practitioners. Possible reasons for this include lower knowledge/awareness about the seriousness of the disease, fear of side effects of medical practitioner’s prescribed treatment, and the associated costs of attending medical appointments (22). Furthermore, there is a widespread myth among illiterate people that chronic illnesses like leprosy and diabetes are ordained by God and the results of bad ‘karma’, and that medical treatments will therefore not be effective in managing these conditions (23).

### 1.2 Physical activity

It is well-known that physical activity plays a vital role in the management of diabetes and its complications by improving various risk factors such as increased weight, blood pressure, cholesterol, stress, and anxiety (24–26). Furthermore, physical activity helps to reduce insulin resistance which is a feature of Type 2 diabetes (27). Despite its physical geography, evidence suggests there is a high burden of physical inactivity (43%), particularly in the urban and semi-urban areas of Nepal (28). Education appeared to play a negative role in one study showing physical inactivity to be three times more common in individuals with higher education levels compared to those with lower education levels (28), possibly due to less educated people carrying out more physically demanding activities such as manual labor in the farm or walking to work. Moreover, it is possible that computer and mobile use, which are a form of sedentary behavior, are fast replacing leisure activities (e.g., field games, social events) amongst the more educated young Nepali adults living in urban areas (29).

### 1.3 Diet

A balanced diet plays a major role in the management of diabetes and the prevention of its complications (30). Consuming food rich in whole grains (such as brown rice, millet, buckwheat), fruits, leafy vegetables, and eating lower amounts of refined grains, red or processed meat, and sweets can help to control blood glucose, cholesterol and blood pressure levels (31). Typical Nepali meals are consumed twice a day and comprise lentil soup (locally known as ‘*daal*’), white rice, and curry rich in purified fat. A meal without rice is regarded as an incomplete meal in Nepali culture (32). White rice is rich in glycemic index, which means its consumption can lead to increased blood glucose levels (33).

Food is central to many festivals in Nepal, such as *Dashain, Tihar, Shivaratri*, *Navaratri*, in which white rice, fat-rich curry, and sweets are served and consumed in large portions. Cultural traditions also play a part; for example, after giving birth to a child, mothers in Nepal are commonly fed three large meals rich in white rice, purified butter, and red meat daily. This is driven by the belief that these foods will keep them strong and warm, which can potentially put them at higher risk of diabetes or its complications.

A study from Kathmandu valley found that 34% of the patients attending a diabetic clinic were not aware that a controlled diet can reduce the risk of diabetic complications (34). Another study from the far western region of Nepal reported that over 87% of diabetic patients attending hospital outpatient department services were practicing poor dietary habits, and ignoring the advice given by health care providers (16).

### 1.4 Gaps in evidence

Target-driven home activities have been found to reinforce healthy changes in the self-care of diabetic patients (35). These activities can include eating a balanced diet and doing regular physical activity with specific, time-bound, and achievable goals (36). Home activities are generally assigned by health care providers and carried out by the patient at home (35). In Nepal, target-driven home activities to promote the healthy control of diabetes are rarely prescribed or practiced. Educational programs aimed at improving knowledge or awareness and practice about diabetes control are usually delivered through workshops, seminars, radio and television interviews, street marches by health professionals, and using pamphlets and flyers (37). Diabetic patients may receive additional information when they visit health care providers, although such information is limited owing to brief consultation times. Professional bodies, like the ‘Nepal Diabetes Association’ (37) and the ‘Diabetes and Endocrinology Association of Nepal’ (38), have dedicated websites that provide public information on diabetes and its complications but lack specific information on overcoming cultural and traditional barriers to good diabetes control. Furthermore, some people in Nepal specifically those who are elderly, illiterate, or living in remote areas often do not have access to television, radio, or internet, and are not able to read (45% of the adult population in Nepal (38)). As a result, the uptake of the available information to improve diabetic knowledge, awareness and practice may vary and is not targeted, and hence may not be optimally effective. Not being able to read and write is a major barrier to having improved knowledge and awareness about diabetes and diabetic retinopathy (39). A recent cross-sectional study of 37094 diabetic patients pooled from 55 low-and middle-income countries, including Nepal, found that fewer than one in ten patients were receiving both the pharmacologic and non-pharmacological interventions including structured educational programs on diabetes self-management (40).

### 1.5 Aim and objectives of the study

This study aimed to address the above gaps and other cultural and traditional barriers of diabetes control identified in previous research (5, 18, 30, 33, 41). A culturally and linguistically appropriate, patient-centered, target-driven lifestyle intervention with multidisciplinary video education training appropriate to local culture and tradition was developed in Nepali language and its effectiveness in improving clinical outcomes, health literacy, and self-care practice pertaining to diet, physical activity-specific changes in newly diagnosed diabetic patients was examined.

The target-driven home-based activity aimed to reduce food portion size, intake of fat, salt, and sweets whilst increasing consumption of locally available healthy food and physical activity incrementally over a period of three months. Participants’ compliance to the intervention was monitored weekly *via* telephone calls. The video training program covered various themes of good diabetes control. This included the uptake of health services, advice on Nepali food, physical activity, repelling myths, retinal screening, patient’s experience of living with diabetes, and a message from a faith healer emphasizing why it is necessary to seek medical help early if someone has got diabetes. The hypothesis that the intervention/training program is significantly effective in improving clinical outcomes of diabetes control, knowledge and awareness, attitude, and practice surrounding diabetes, as well as promoting the uptake of diabetic retinopathy screening was examined over a period of three months.

## 2 Method

### 2.1 Study type

A single-blind two-arm randomized controlled study (longitudinal over three months) in which the clinical outcome measurements were blinded from the randomization and intervention allocations. Data were collected between May 2019 and March 2020.

### 2.2 Study design

Two (participant groups: intervention and control) × two (visits: baseline and 3-month follow-up) mixed design.

### 2.3 Setting

This study was conducted at the Department of Medicine, Gandaki Medical College and Teaching Hospital, Pokhara, Nepal in collaboration with the Vision and Eye Research Institute, Anglia Ruskin University, Cambridge, UK. The study protocol was approved by Nepal Health Research Council (Reference no. 2167) and was registered as a clinical trial (ISRCTN10990062).

### 2.4 Participants

A total of 110 participants with newly diagnosed diabetes and fasting blood sugar level ≥126 mg/dL were invited to take part in the study. They were randomly assigned into two groups: those receiving training on the patient-centered intervention program (n = 55, intervention group) and the control group (n = 55, control group). The intervention was co-developed with advice from patients with diabetes who were neither included in the study nor in the interpretation or writing of findings.

The following inclusion criteria were applied: (i) adult participants above 18 years of age with newly diagnosed Type 2 diabetes; (ii) participants willing to take part in the study; (iii) participants able to provide informed written or verbal consent. Exclusion criteria were: (i) participants self-reporting to have significantly impaired memory functions and other conditions that affect the capacity to give consent such as dementia, stroke, Huntingdon’s disease, etc.; (ii) participants with significant mobility issues; (iii) participants who were unable to provide informed consent; (iv) more than one participant from the same family.

Participants were treated in accordance with the applicable ethical guidelines that followed the tenets of the Helsinki Declaration. Information that could identify individual participants during or after data collection was not recorded. Participants were assured that they did not have to take part in the study or if they wished to drop out of the study once they decided to take part, it would not affect any care or treatment they would otherwise receive from their doctor or hospital. All participants took their prescribed treatment from the doctor regardless of which group they belonged to.

### 2.5 Sample size calculation

To calculate the sample size, we used 0.88 standard deviation of the changes in HbA1c levels following health interventions in South Asian diabetic patients (42), with a minimum clinical difference of 0.52. For a two-sample, two-sided t-test at the 5% significance level with a power of 80%, the minimum required number of participants for each group was calculated to be 45 (43). Taking into account some attrition, we aimed for a total sample size of 55 participants in each group. The proposed sample size is higher than the 42 patients in a clinical trial that used a 12-week intervention duration similar to the current study to compare the effects of different exercise programs in patients with Type 2 diabetes (44).

### 2.6 Study material- questionnaire

A semi-structured questionnaire provided in **Supplementary Table 1** was used to obtain information about demographic and knowledge and awareness about diabetes and its control. The questionnaire was built on an existing, validated questionnaire (45) and previously published findings (19). The questionnaire was translated into Nepali and then independently translated back to English. After that, both versions were checked to examine whether any revisions of the translation were required. The questionnaire was pretested on a small sample of 15 people with diabetes who were not included in the study.

### 2.7 Procedure

Eligible participants were identified for their suitability to be included in the study by a trained data collector (research assistant) under the supervision of a diabetic clinician (author TLU). Participants meeting the inclusion criteria were allocated into ‘intervention’ and ‘control’ groups by simple randomization method, using random sequence of computer-generated numbers from 1-110 each representing participant number. The doctor who provided both groups with their medication and usual care was masked from participant allocation. All participants provided informed consent for taking part in the study. Baseline data on age, gender, diet, physical activity, diabetic knowledge/awareness, attitude, and practice, HbA1c, total cholesterol, high-density lipoprotein (HDL), low-density lipoprotein (LDL), blood pressure, height, weight, and visual acuity as a log of the minimum angle of resolution (logMAR) were collected from participants in both groups. Questionnaire data were collected by the research assistant. Clinical data were obtained by the treating clinician, and both were blinded to each other’s findings. After the baseline data collection, only those in the intervention group were trained on a single-session multidisciplinary diabetic education program that constituted short video clips in the Nepali language developed following a brainstorming session with a multidisciplinary team of experts (**Supplementary Table 2**). The video education training lasted approximately 12 minutes with brief intervals provided in between, during which participants could ask questions if they did not understand any aspect of the video program.

Data collection on knowledge/awareness was repeated immediately after the training for those participants in the intervention group to ascertain whether participants understood the information provided in the video. A dietary recall questionnaire was used to assess dietary intake during the previous 24 hours in order for a dietician to develop a customized diet plan for each participant in the intervention group. Levels of physical activity were also ascertained. A patient-centered dietary and physical activity intervention with specific targets to practice at home, together with a logbook for keeping a daily record of their diet and physical activity routine, was developed as provided in **Supplementary Table 3**. For illiterate participants, their family members helped with the recording. Participant’s compliance with the recommended intervention was monitored weekly *via* telephone calls. All 110 participants were followed up after three months.

The outcome measures compared after 3 months were HbA1c, cholesterol, blood pressure, BMI (weight in kilogram/height in meter squared), visual acuity, knowledge/awareness, and practice about diabetes, and attendance at diabetic retinopathy screening.

### 2.8 Data analysis

Clinical data such as HbA1c, total cholesterol, blood pressure, and BMI were analyzed in SPSS using two (participant groups) × two (visits) mixed analysis of variance (ANOVA) with Bonferroni corrected applied. Questionnaire data were compared between the participant groups using a chi-square (χ^2^) test (Fisher’s exact).

## 3 Results

The mean age of participants in the intervention and the control groups were 45 ± 10 years and 47 ± 12 years respectively with no significant difference between the groups (*p* = 0.23). There were 27 (49.1%) males and 28 (50.9%) females in each group. Over 20% of participants in each group were illiterate and the numbers did not differ significantly between the groups. Examination of the logbook record at the 3-month follow-up visit confirmed that all participants had met specific targets of physical activity and diet interventions suggested to them. All participants in both groups attended the follow-up visit. Almost all (98.2%) participants in the intervention group reported that the information provided in the diabetic education video training program was moderate to very easy to understand.

### 3.1 Clinical data

Mean HbA1c, total cholesterol, HDL, LDL, body mass index, systolic blood pressure, diastolic blood pressure, and log MAR visual acuity at the baseline and 3-month follow-up visit are provided in **Table 1**.

**Table 1:** Mean ( ± standard deviation) for clinical variables measured at baseline and 3-month follow-up visit for participants in the intervention and the control group along with the results of 2 (participant groups) *2 (visits) ANOVA and the Bonferroni corrected *post hoc* comparisons.

Clinical variable	Visit	Group	Mean (±SD)	2*2 mixed ANOVA: Main effects	p-value	Posthoc comparisons-Bonferroni corrected		p-value
HbA1c (%)	Baseline	Intervention	7.2 (±1.5)	Participant group, F(1,108)=0.4	0.53	Intervention	(Baseline vs. 3-month)	<0.001
		Control	7.1 (±1.8)	Visit, F(1,108) = 30.3	<0.001	Control	(Baseline vs. 3-month)	0.02
	3-month	Intervention	6.1 (±1)	Participan*visit interaction,	0.04	Baseline	(Intervention vs. control)	0.61
		Control	6.6 (±1.2)	F(1,108) = 4.5		3-month	(Intervention vs. control)	0.03
Total cholesterol (mg/dL)	Baseline	Intervention	184.1 (±44.4)	Participant group, F(1,101)=0.01	0.99	Intervention	(Baseline vs. 3-month)	0.07
		Control	172.6 (±27.8)	Visit, F(1,101)=0.2	0.65	Control	(Baseline vs. 3-month)	0.02
	3-month	Intervention	174.3 (±33.2)	Participan*visit interaction,	0.003	Baseline	(Intervention vs. control)	0.12
		Control	185.9 (±33.4)	F(1,101)=9.2		3-month	(Intervention vs. control)	0.08
Triglycerides (mg/dL)	Baseline	Intervention	161.9 (±85.3)	Participant group, F(1,101)=0.16	0.7	Intervention	(Baseline vs. 3-month)	0.66
		Control	156.1 (±89.4)	Visit, F(1,101)=0.32	0.58	Control	(Baseline vs. 3-month)	0.73
	3-month	Intervention	156.6 (±65.3)	Participan*visit interaction,	0.95	Baseline	(Intervention vs. control)	0.74
		Control	152 (±76)	F(1,101)=0.004		3-month	(Intervention vs. control)	0.74
High density lipoprotein (HDL) (mg/dL)	Baseline	Intervention	47 (±6.3)	Participant group, F(1,101)=3.82	0.05	Intervention	(Baseline vs. 3-month)	0.001
		Control	45.1 (±5.7)	Visit, F(1,101)=19.8	<0.001	Control	(Baseline vs. 3-month)	0.003
	3-month	Intervention	50.3 (±6.6)	Participan*visit interaction,	0.87	Baseline	(Intervention vs. control)	0.12
		Control	48.2 (±6.8)	F(1,101)=0.03		3-month	(Intervention vs. control)	0.11
Low density lipoprotein (LDL) (mg/dL)	Baseline	Intervention	99.6 (±25)	Participant group, F(1,96)=1.37	0.25	Intervention	(Baseline vs. 3-month)	0.28
		Control	97.6 (±25.6)	Visit, F(1,96)=1.2	0.27	Control	(Baseline vs. 3-month)	0.01
	3-month	Intervention	95.5 (±22.3)	Participan*visit interaction,	0.01	Baseline	(Intervention vs. control)	0.69
		Control	107.5 (±26)	F(1,96)=6.97		3-month	(Intervention vs. control)	0.02
Body Mass Index (BMI)	Baseline	Intervention	28.2 (±3.7)	Participant group, F(1,107)=0.44	0.51	Intervention	(Baseline vs. 3-month)	0.08
		Control	28.4 (±4.4)	Visit, F(1,107)=1.17	0.28	Control	(Baseline vs. 3-month)	0.84
	3-month	Intervention	27.6 (±5.2)	Participan*visit interaction,	0.17	Baseline	(Intervention vs. control)	0.82
		Control	28.5 (±4.2)	F(1,107)=1.87		3-month	(Intervention vs. control)	0.33
Systolic blood pressure (mmHg)	Baseline	Intervention	125.7 (±13.5)	Participant group, F(1,104)=0.58	0.45	Intervention	(Baseline vs. 3-month)	0.92
		Control	122.3 (±13.4)	Visit, F(1,104)=1.57	0.21	Control	(Baseline vs. 3-month)	0.06
	3-month	Intervention	125.5 (±11.7)	Participan*visit interaction,	0.17	Baseline	(Intervention vs. control)	0.20
		Control	125.7 (±12)	F(1,104)=1.96		3-month	(Intervention vs. control)	0.94
Diastolic blood pressure (mmHg)	Baseline	Intervention	83.8 (±7.9)	Participant group, F(1,104)=3.47	0.07	Intervention	(Baseline vs. 3-month)	0.14
		Control	81.1 (±8.5)	Visit, F(1,104)=3.51	0.06	Control	(Baseline vs. 3-month)	0.25
	3-month	Intervention	82.1 (±7.7)	Participan*visit interaction,	0.82	Baseline	(Intervention vs. control)	0.10
		Control	79.8 (±7.7)	F(1,104)=0.06		3-month	(Intervention vs. control)	0.13
LogMAR visual acuity-Right Eye	Baseline	Intervention	0.04 ((±0.08)	Participant group, F(1,105)=0.13	0.72	Intervention	(Baseline vs. 3-month)	0.62
		Control	0.05 (±0.01)	Visit, F(1,105)=0.12, p=0.73	0.73	Control	(Baseline vs. 3-month)	0.99
	3-month	Intervention	0.04 (±0.08)	Participan*visit interaction,	0.73	Baseline	(Intervention vs. control)	0.80
		Control	0.05 (±0.1)	F(1,105)=0.12		3-month	(Intervention vs. control)	0.66
LogMAR visual acuity- Left Eye	Baseline	Intervention	0.04 (±0.08)	Participant group, F(1,105)=0.07	0.79	Intervention	(Baseline vs. 3-month)	0.97
		Control	0.04 (±0.09)	Visit, F(1,105)=0.21	0.65	Control	(Baseline vs. 3-month)	0.55
	3-month	Intervention	0.04 (±0.08)	Participan*visit interaction,	0.69	Baseline	(Intervention vs. control)	0.93
		Control	0.05 (±0.1)	F(1,105)=0.16		3-month	(Intervention vs. control)	0.69

**Figure 1:**
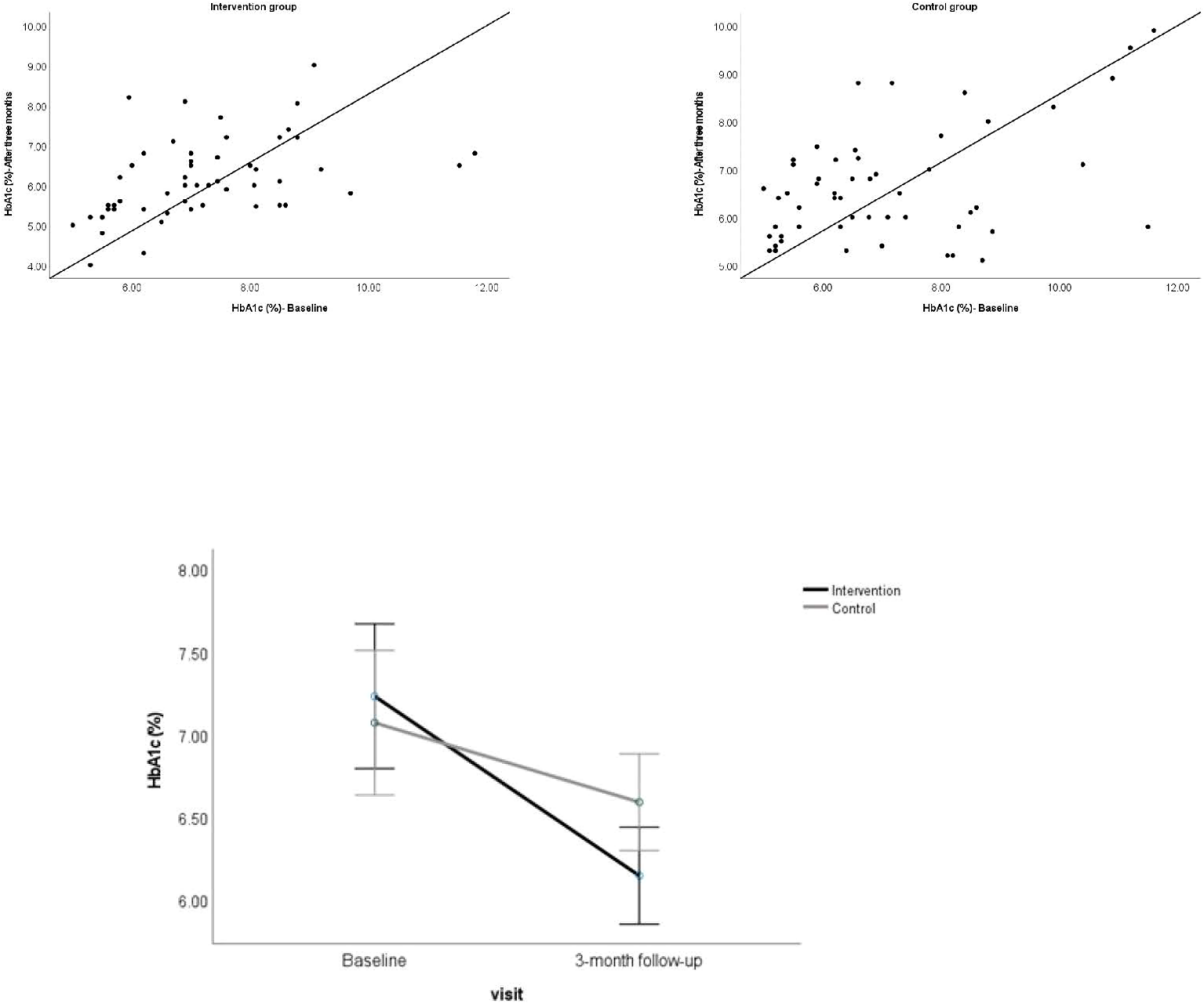
Above. Scatterplot showing the HbA1c for individual participants at baseline against 3-month follow-up visit. Below. Showing the mean HbA1c for the participant groups at baseline and 3-month follow-up visit. Error bars represent 95% CI.

Overall, in 92.7% of the participants in the intervention group and 60% of those in the control group, the HbA1c level was found to decrease from the baseline to three months follow-up visit. In the intervention group, the mean HbA1c averaged across the participants decreased from 7.2% at baseline to 6.1% at the 3-month visit. In the control group, it decreased from 7.1% at baseline to 6.6% at 3-month visit. A two (participant groups) × two (visits) mixed ANOVA on HbA1c data showed a significant main effect for visit, but not for participant group (**Table 1**). A significant interaction between the effects of the participant group (intervention and control) and the type of visit (baseline and follow-up) on HbA1c was found (**Table 1**, **Figure 1**), showing that the reduction in mean HbA1c level from baseline to 3-month visit was significantly greater for participants in the intervention group compared to those in the control group.

**Figure 2:**
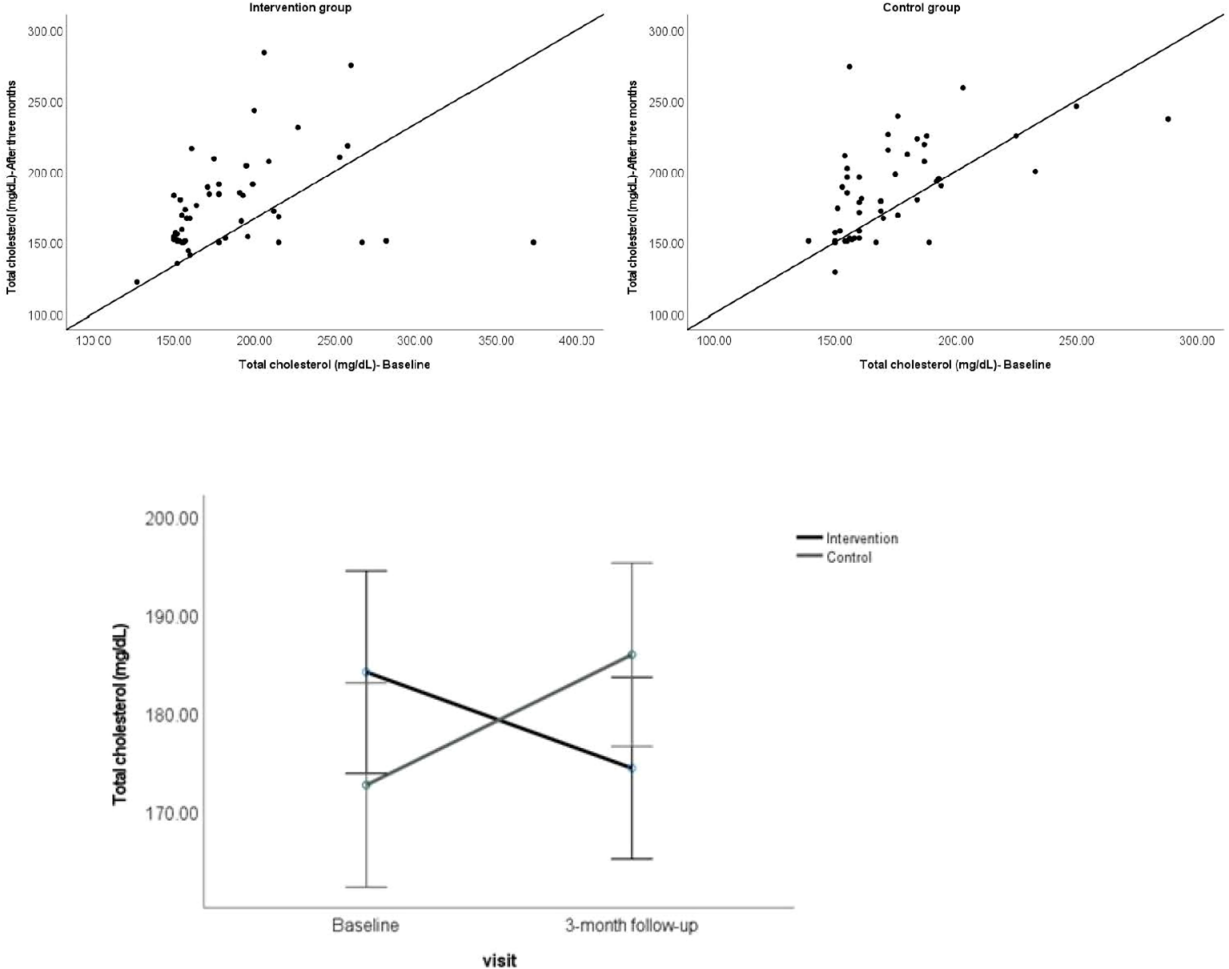
Above. Scatterplot showing the total cholesterol for individual participants at baseline against 3-month follow-up visit. Below. Showing the mean total cholesterol for the participant groups at baseline and 3-month follow-up visit. Error bars represent 95% CI.

A two (participant groups) × two (visits) mixed ANOVA on total cholesterol did not show a significant main effect of the participant group and visit, but a significant interaction between effects of participant group and visit type was found (**Table 1**, **Figure 2**), showing that the change in mean total cholesterol from baseline to 3-month follow-up visit differed significantly between the participants groups.

**Figure 3:**
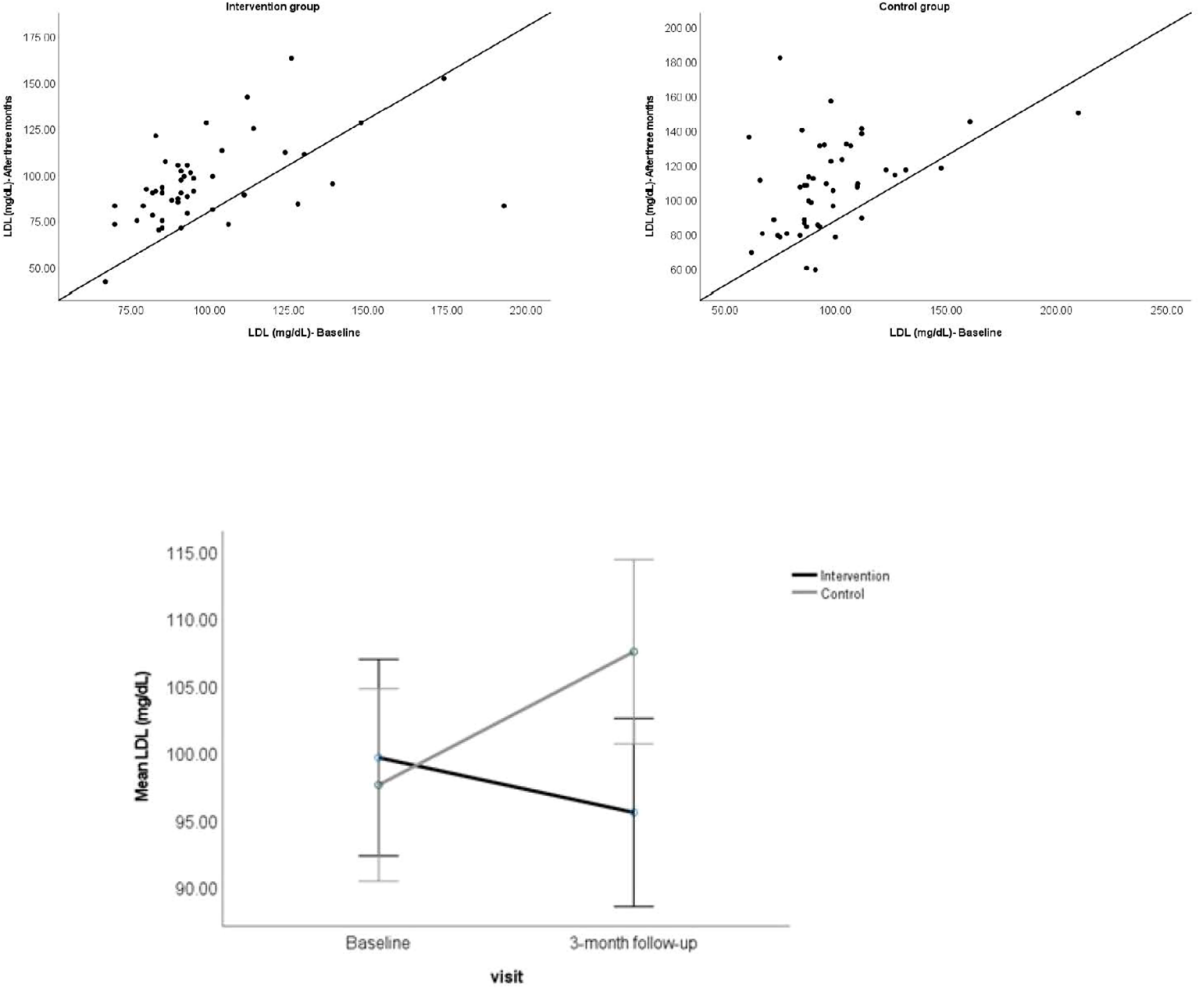
Above. Scatterplot showing the LDL for individual participants at baseline and 3-month follow-up visit. Below. Showing the mean LDL for the participant groups at baseline and 3-month follow-up visit. Error bars represent 95% CI.

A significant interaction between the effects of the participant group (intervention and control) and the type of visit (baseline and 3-month follow-up) was also found for LDL levels, *F (1*,96) = 6.97, *p* = 0.01 (**Table 1**, **Figure 3**).

The mean HDL between the baseline and 3-month visit did not differ significantly between the participant groups, nor did other clinical variables including log MAR visual acuity, BMI, and systolic and diastolic blood pressure.

### 3.2 Questionnaire data

#### 3.2.1 Baseline data on knowledge/awareness about diabetes and DR

Baseline data showed that participants had poor knowledge and awareness about diabetes and DR. Out of all 110 participants, 45.5% did not believe that diabetes is a serious disease, and 39.2% and 31.8% did not know that good cholesterol and blood pressure levels were important in controlling diabetes, respectively. Of the total, 76.4% of participants did not know the correct fasting blood glucose (≥126 mg/dL) or HbA1c level (>6.5%) for diagnosing diabetes. Of the total, 37.3% did not know that stress can cause diabetes, 12.7% thought that sitting down will help to control diabetes, and 61.8% did not know that there was a difference between having eyes tested for glasses and diabetes. Out of the total, 32% of participants did not know that diabetes can affect the eyes, and none (0%) knew that photograph of the back of the eye is taken to test if diabetes has affected the eyes.

#### 3.2.2 Baseline data on attitude and practice

Almost all participants (98.2%) thought that it would be difficult to eat a healthy diet during festivals and weddings. On average, participants reported consuming a high amount of carbohydrates daily (nearly 2 cups of white rice every day, >300 grams). Around 5% said they smoked a cigarette and 20% said they drank alcohol.

None of the baseline data on knowledge/awareness, attitude, and practice differed significantly between the participant groups (*p >*0.05).

#### 3.2.3 Change in knowledge/awareness, and attitude immediately after training on the video education program

Data collected immediately after the video education training for the participants within the intervention group confirmed that the training had significantly improved knowledge/awareness about diabetes and its control wherein a significantly greater number of participants after the training vs. before correctly answered the following questions: (i) is good blood pressure control important for controlling diabetes?; (ii) is good cholesterol control important for controlling diabetes?; (iii) do you think stress can cause diabetes?; (iv) do you know what level of blood sugar is normal to suggest non-diabetes?; (v) can diabetes affect eyes?; (vi) do you think there is a difference between having your eyes tested for spectacles and for diabetes?; (vii) what test is usually done to check if diabetes has affected your eyes?; (viii) would you see a doctor regularly to have your eyes tested even if you were seeing well?; (ix) would you see a doctor regularly even if you think your diabetes is well controlled? (**Table 2**).

**Table 2:** Percentage of participants in each response category of the questionnaire data in the intervention group before vs. immediately after training on the video education program.

Question	Participant response	For intervention group only (% of participants)		p-value
		Before the workshop	Immeditely after the workshop	
Is good BP control important for controlling diabetes?	yes	69.10%	100%	<0.001
	no/not sure	30.90%	0%	
Is good cholesterol control important for controlling diabetes?	yes	61.80%	100%	<0.001
	no/not sure	39.20%	0%	
Do you think diabetes is a serious disease?	yes	63.60%	76.40%	0.21
	no/not sure	36.40%	23.60%	
What level of blood sugar is normal to suggest non-diabetes?	Fasting <126 mg/dL, HbA1c ≤6.5%	30.90%	69.10%	<0.001
	not sure	69.10%	38.90%	
Would you see doctor regularly even if you think your diabetes is well controlled?	yes	89.10%	100%	<0.001
	no/not sure	10.90%	0%	
Do you think stress can cause diabetes or make it worse?	yes	78.20%	92.70%	0.06
	no/not sure	21.80%	7.30%	
Do you think that medication you have been given by doctor can be replaced by herbal/traditional medicine?	yes	10.90%	1.80%	0.12
	no	89.10%	98.20%	
How often would you check your blood sugar?	Every three months	89.10%	100%	0.03
	Every year/not sure	10.90%	0%	
Do you think eating mithai (sweets) is bad for your diabetes?	yes	90.90%	98.20%	1
	no/not sure	9.10%	1.80%	
Do you think it would be difficult to eat healthy diabetic diet during festivals and weddings?	yes	100%	100%	0.24
	no	0%	0%	
Do you do think sitting down will help to control diabetes?	yes	10.90%	3.60%	0.27
	no/not sure	89.10%	96.40%	
Do you think activity like going to farm, walking in hills help to control your diabetes?	yes	90.90%	100%	0.01
	no/not sure	9.10%	0%	
Can diabetes affect eyes?	yes	81.80%	100%	0.006
	no/not sure	18.20%	0%	
Do you think there is a difference between having your eyes tested for spectacles and for diabetes?	yes	40%	72.70%	<0.001
	no/not sure	60%	27.30%	
Do you know what test is usually done to check if diabetes has affected your eyes?	yes	0%	67.30%	<0.001
	no/not sure	100%	32.70%	

P-values were calculated using chi-square test.

#### 3.2.4 Change in knowledge/awareness and attitude about diabetes and DR after three months

The improved knowledge and awareness about diabetes and its control in the intervention group as a result of being trained in the video-based education program was found to be sustained, or even improved, after three months. The control group showed a non-significant improvement from the baseline to the 3-month follow-up visit for all the variables, with a slight but non-significant decrease from the baseline in the number of participants who reported knowing the correct blood sugar levels for diagnosing diabetes. After three months, percentage of participants who were aware that the control of blood pressure was important in controlling diabetes increased by 30.9% for the intervention group, compared to just 3.6% in the control group; this difference was statistically significant (*p <*0.05). Likewise, the percentage increase after three months (from the baseline) in the intervention group vs. the control group was statistically significant who correctly knew the following: (i) control of blood pressure is important in controlling diabetes; (ii) control of cholesterol is important in controlling diabetes; (iii) diabetes is a serious disease; (iv) fasting blood glucose level of ≥126 mg/dL or HbA1c level of ≥6.5% is classified as diabetes; (v) stress is an important risk factors for diabetes; (vi) physical activities like working in the going to farm, walking in hills can help to control diabetes; (vii) diabetes can affect eyes; (viii) there is a difference between having eyes tested for spectacles and for diabetes, and that screening for diabetic eye disease is carried out by taking photographs of the back of the eyes. Also, a significantly greater percentage of participants in the intervention group vs. the control group after three months said that they would check their blood sugar levels at least once every three months and would go to see an eye doctor regularly even if they were seeing well, as shown in **Table 3**.

**Table 3:** Percentage of participants in each response category of the questionnaire data at baseline and 3-month follow-up visit for participants in the intervention and the control group.

Question	Participant response	Baseline			3-months follow-up visit		
		Intervention	Control	p-value	Intervention	Control	p-value
Is good BP control important for controlling diabetes?	yes	69.10%	67.30%	0.99	100%	70.90%	<0.001
	no/not sure	30.90%	32.70%		0%	29.10%	
Is good cholesterol control important for controlling diabetes?	yes	61.80%	61.80%	0.99	100%	72.70%	<0.001
	no/not sure	39.20%	39.20%		0%	27.30%	
Do you think diabetes is a serious disease?	yes	63.60%	45.50%	0.08	92.70%	47.30%	<0.001
	no/not sure	36.40%	54.50%		7.30%	52.70%	
Do you know what level of blood sugar is normal to suggest non-diabetes?	Fasting <126 mg/dL, HbA1c <6.5%	30.90%	16.40%	0.12	85.50%	9.10%	<0.001
	no/not sure	69.10%	83.60%		14.50%	89.90%	
Do you think stress can cause diabetes or make it worse?	yes	78.20%	65.50%	0.2	96.40%	70.90%	<0.001
	no/not sure	21.80%	34.50%		3.60%	29.10%	
Do you think medication you have been given by doctor can be replaced by herbal medicine/traditional healers?	yes	10.90%	20%	0.99	3.60%	14.50%	0.09
	no	89.10%	80%		96.40%	85.50%	
How often would you check your blood sugar levels?	At least once every three months	89.10%	90.90%	1.00	100%	89.10%	0.03
	Every year/not sure	10.90%	9.10%		0%	10.90%	
Should you see a doctor regularly even if you think your diabetes is well controlled?	yes	89.10%	98.20%	0.11	100%	100%	not calculated
	no/not sure	10.90%	1.80%		0%	0%	
Do you think it would be difficult to eat a good diabetic diet during festivals and weddings?	yes	100%	96.40%	0.49	100%	94.50%	0.24
	no/not sure	0%	3.60%		0%	5.50%	
Do you think eating mithai (sweets) is bad for your diabetes?	yes	90.90%	98.20%	0.21	98.20%	96.40%	1
	no/not sure	9.10%	1.80%		1.80%	3.60%	
Do you think sitting down will help to control diabetes?	yes	10.90%	14.50%	0.78	3.60%	10.90%	0.27
	no/not sure	89.10%	85.50%		96.40%	89.10%	
Do you think activity like going to farm, walking in hills help to control your diabetes?	yes	90.90%	87.30%	0.76	100%	87.30%	0.01
	no/not sure	9.10%	12.70%		0%	12.70%	
Is smoking bad for diabetes?	yes	92.70%	89.10%	0.74	100.0%	94.50%	0.24
	no/not sure	4 7.3%	10.90%		0.00%	5.50%	
Is drinking alcohol bad for diabetes?	yes	20.00%	20.00%	1	95%	89.10%	0.49
	no/not sure	80.00%	80.00%		6%	10.90%	
Can diabetes affect eyes?	yes	81.80%	85.50%	0.5	100%	85.50%	0.006
	no/not sure	18.20%	14.50%		0%	14.50%	
Do you think there is a difference between having your eyes tested for spectacles and for diabetes?	yes	40%	36.40%	0.84	87.30%	43.60%	<0.001
	no/not sure	60%	63.60%		12.70%	56.40%	
Do you know what test is usually done to check if diabetes has affected your eyes?	yes	0%	0%	not calculated	87.30%	3.60%	<0.001
	no/not sure	100%	100%		12.70%	96.40%	
Would you go to see an eye doctor to have your eyes tested even if you were seeing well?	yes	36.40%	30.90%	0.69	100%	40%	<0.001
	no	63.50%	69.10%		0%	60%	

P-values were calculated using chi-square test. List of supplementary tables.

#### 3.2.5 Change in practice after three months

After three months, the amount of daily white rice consumption decreased by 36.5% (from the baseline) in the intervention group compared to just 4% in the control group, and the difference was statistically significant (*p <*0.05). All participants (100%) in the intervention group had the DR screening done as confirmed by their ophthalmological report where 13% were reported to have early signs of DR, compared to 0% in the control group. At the baseline visit, participants in the intervention group reported doing daily physical exercise of 0.91 ± 0.49 hours compared to 0.89 ± 0.52 hours in the control group, and the difference was not significant. However, at the 3-month follow-up visit, the number of hours of daily exercise done was significantly greater for the intervention group (1.03 ± 0.48) compared to the control group (0.82 ± 0.51) (*p <*0.05). When asked how many hours of sitting down do you do every day, participants in the intervention group reported 4.1 ± 2.1 hours at baseline compared to 4.8 ± 1.9 in the control group, and the difference was not significant. However, at the 3-month follow-up visit, the number of hours of sitting down each day was significantly lower for the intervention group (3.8 ± 1.8) compared to the control group (4.7 ± 2.2) (*p <*0.05).

## 4 Discussion

Management of diabetes and its complications has become a major challenge for most countries. Lack of availability of information including a lack of a culturally appropriate education program, poor health literacy, poor compliance to treatment, and poor attendance to health care appointments all play a major role (18, 46–50). This study is the first randomized controlled trial to design and evaluate the effectiveness of a culturally and linguistically appropriate patient-centered target-driven lifestyle intervention with video education training to improve clinical outcomes of diabetes control, health literacy, and self-care practice in newly diagnosed diabetic patients in Nepal. The lifestyle intervention aimed at achieving specific and measurable goals for the types and portion of foods eaten as well as increasing physical activity steadily over a period of three months. Participants in the intervention group were monitored weekly *via* telephone calls to ensure that they were complying with the suggested interventions. The video education training at the baseline visit aimed to improve patients’ knowledge and awareness about the disease and motivate them to adhere to the recommended practice-based diet and physical activity interventions at home. The intervention was culturally appropriate as it improved awareness around the factors that influence people’s habits in Nepal, including the consumption of sweets during festivals and seeking help from faith healers.

Our first hypothesis was that participants who were trained in the intervention program present with significantly improved control of diabetes at the 3-month follow-up visit. The findings supported this hypothesis. The reduction in mean HbA1c levels from the baseline to the 3-month visit in the intervention group was significantly greater than in the control group. It is worth mentioning that mean HbA1c levels decreased significantly after three months from the baseline visit for both groups of participants, but the decline in HbA1c, from baseline to the 3-month visit was significantly higher for the intervention group (from 7.2% to 6.1%) compared to the control group (from 7.1% to 6.6%. The mean HbA1c level of 6.1% found in the intervention group after three months is below the recommended threshold value of 6.5%. However, the mean HbA1c level was 6.6% in the control group suggesting that the HbA1c levels were not adequately controlled at the three months follow-up visit in this group. Although our multidisciplinary intervention was found to be effective in controlling the total cholesterol levels, the effect of each of the individual components could not be ascertained with statistical certainty and is part of our future study with a larger number of participants. After three months, LDL levels reduced by 4.1% from the baseline value of 99.6 mg/dL to of 95.6 mg/dL in the intervention group, which was significantly lower than 107.5 mg/dL (an increase of 10%) in the control group. Clinical threshold values of <100 mg/dL LDL in adults are accepted as ‘within the normal range.’ A previous trial from the US called ‘action for health in diabetes’ evaluating the effects of an intensive lifestyle intervention (calorie control and increased physical activity for weight loss) in overweight and obese patients with Type 2 diabetes found that HbA1c levels decreased significantly by 8.3% after one year (51). A study from Denmark examining the effect of a lifestyle intervention (aerobic exercise combined with the diet control plan) on glycaemic control in people with longstanding Type 2 diabetes of up to 10 years duration found that, from baseline to 12‐month follow‐up, the mean HbA1c level changed from 6.65% to 6.34% in the intervention group and from 6.74% to 6.66% in the standard care group, but with a non-significant statistical difference between the participant groups, *p* = 0.14 (52).

Although systolic and diastolic blood pressure decreased, and HDL levels increased from baseline to three months in the intervention group, when compared to the control group, the differences were not found to be statistically significant. This was a surprising finding especially since the other parameters such as HbA1c data were significantly reduced. Fasting blood sugar and blood pressure readings tend to be affected by various parameters which are outside the control of the data collector, such as the size and content of the last meal patient had consumed and sodium intake (53). Other clinical data such as HbA1c are designed to be averaged across a longer period. It is also possible that the non-significant differences may be because of the relatively short follow-up period (three months) used in the current study and our future follow-up study would look at that in more detail. Overall, participants in our study were found to have poor knowledge/awareness and practice about diabetes at the baseline visit confirming previous findings (17, 18, 30). None of the baseline data differed significantly between the participant groups, i.e., who were trained vs. not trained in the diabetic education program. Our second hypothesis was that the video training program was significantly effective in improving knowledge/awareness, attitude, and practice about diabetes, and in promoting the uptake of diabetic retinopathy screening. The findings largely supported this hypothesis. Within the intervention group, a comparison of data before vs. immediately after the training showed that the diabetic education program was significantly effective in improving knowledge, awareness, and practice about diabetes control. Diabetic health literacy improved significantly for the intervention group, both, from (the baseline before training) and from the control group after three months supporting our second hypothesis. For example, a significantly greater percentage of participants in the intervention group at the follow-up visit knew that diabetes is a serious disease, a fasting blood glucose level of ≥126 mg/dL or HbA1c level of >6.5% is classified as diabetes, stress can cause diabetes, diabetes can affect eyes, and activities like farming or walking in hills can help to control diabetes.

Also, participants in the intervention group were found to show evidence of improved diabetic self-care practice after three months, in which they self-reported less consumption of white rice and increased physical activity compared to those in the control group.

Our study, however, also shows that participants from both groups would find it difficult to resist sweets and sugary food during festivals and social ceremonies. This agrees with previous studies reporting the importance of cultural beliefs, for example, the meal is not complete without eating rice, and social pressure to consume unhealthy food during festivals or social gatherings (54). These results warrant a need for further interventions to promote positive attitudes around healthier eating, especially during festivals and social ceremonies. This may require more innovative methods, perhaps using role models to promote better behavior, reducing peer pressure, and other types of encouragement and initiatives. Weekly follow-ups and a reminder to keep on the course were very important in this study. We agree this would not be sustainable in the longer term and we are examining how often this needs to be done in order for the results shown to be sustained.

All participants in the intervention group presented a diabetic retinopathy screening report at the 3-month follow-up visit compared to 0% in the control group. A hospital-based retrospective study conducted in Kathmandu valley reported that over 50% of the patients had already developed sight-threatening retinopathy at first presentation (55). Increased health literacy has been found to be associated with improved uptake of health care services (56). Also, a Cochrane review of 33 studies from the low and middle-income countries, published in 2018, suggested that designing a culturally appropriate retinopathy screening intervention might be effective in improving low attendance of DR screening services in these countries (53).

A prior study from Nepal examining the effect of a diet-focussed diabetic education intervention, delivered in the form of PowerPoint presentations at the baseline visit, reported significantly lower calorie intake and improved plasma glucose levels from 8.6 mmol/L to 6.7 (1.2) mmol after six months in those who received the intervention compared to the controls in whom the change was from 8.1 mmol/L to 7.0 mmol/L (57). Our study goes a step further in using a holistic, patient-centered approach which, combined a healthy food intervention, physical activity, and awareness of diabetes and its complications in newly diagnosed patients, who were monitored regularly. Multi-faceted approaches are better in that they deal with a number of factors influencing diabetes. On the other hand, it is not possible to ascertain whether the observed changes in clinical parameters were due to individual interventions (e.g., diet control, physical activity), or a combination of all or some. Either way, a holistic approach is often better than an individual one as many risk factors for diabetes are interrelated.

Findings from the present study must be interpreted in light of some limitations. Double blinding was not possible as participants would know the group they belonged to. The contribution of individual interventions such as diet control, physical activity, and pharmacologic treatment could not be discerned. Nonetheless, our findings suggest that clinical outcomes of diabetes control (more than twice in HbA1c levels) and diabetes knowledge/awareness can be significantly improved in as little as three months in newly diagnosed patients using standard treatment supplemented by a patient-centered diet and physical activity intervention program, and a video-based diabetic education training session provided at the baseline visit. This approach is important as the literature suggests no significant effect of a structured diabetic education intervention (developed in the UK by DESMOND) on improving blood glucose levels after three years (58). Our findings suggest that health care providers should work with allied health professionals, including dieticians and physical activity specialists, to develop a more holistic program that may lead to improved lifestyle and diabetic control in Nepal. A culturally and linguistically appropriate training program using video clips is effective in improving knowledge/awareness, attitude, and practice around diabetes control and promoting retinal screening uptake in Nepal. This may be vital in reducing the risk of diabetic complications including diabetes-related blindness, and in developing a national guideline for diabetes care in Nepal, which is lacking currently.

## Data availability statement

The raw data supporting the conclusions of this article will be made available by the authors, without undue reservation.

## Ethics statement

The studies involving human participants were reviewed and approved by Nepal Health Research Council (Reference no. 2167). The patients/participants provided their written informed consent to participate in this study.

## Author contributions

SP, RS, TL, LS, JK, and BA contributed to the conception and design of the study. ST and TS helped with data collection. SP and RS wrote the main manuscript and did the analyses. All authors contributed to the article and approved the submitted version.
