# Supplementary material for: Individual patient-centered target-driven intervention to improve clinical outcomes of diabetes, health literacy, and self-care practices in Nepal: A randomized controlled trial
Source: Front Endocrinol (Lausanne). 2023 Jan 19;14:1076253. doi: 10.3389/fendo.2023.1076253 (PMC9893775; doi:10.3389/fendo.2023.1076253)
Supplement: Supplementary file 1 [file Table_1.pdf]

Age ……	Gender ……
Marital status ……	Education level…..
Do you think diabetes is a serious disease?	
Do you think it is up to you or your doctor to control your diabetes?	
Do you know what level of blood sugar is normal to suggest non-diabetes?	
Is good blood pressure control important for controlling diabetes?	
Is good cholesterol control important for controlling diabetes?	
Should you see a doctor regularly even if you think your diabetes is well controlled?	
Do you think that the medication you have been given by your doctor can be replaced by ayurvedic medicine or traditional healers?	
Do you think stress can make diabetes worse?	
How often would you check your blood sugar?	
How much rice do you eat a day?	
Do you think sweets ('mithai') is bad for your diabetes?	
Do you think it would be difficult to eat a good diabetic diet during festivals and weddings?	
How many hours of physical exercise such as walking or running do you do every day?........................	
Do you think that everyday activities like going to the farm, and walking up and down the hills, will help in controlling your diabetes?	
How many hours of sitting down do you do a day? ……………………	
Do you think that sitting down will help you control your diabetes?	
Do you smoke?	
Is smoking bad for diabetes?	
Do you drink alcohol?	
Is drinking alcohol bad for diabetes?	
Can diabetes affect the eyes?	
Do you think there is a difference between having your eyes tested for spectacles and for diabetes?	
Have you had a diabetic eye check-up from a doctor?	
Do you know what test is done to check if diabetes has affected your eyes?......	
Would you go to an eye doctor to have your eyes tested even if you were seeing well?	

Supplementary Table 1. Questionnaire used
