# Supplementary material for: Individual patient-centered target-driven intervention to improve clinical outcomes of diabetes, health literacy, and self-care practices in Nepal: A randomized controlled trial
Source: Front Endocrinol (Lausanne). 2023 Jan 19;14:1076253. doi: 10.3389/fendo.2023.1076253 (PMC9893775; doi:10.3389/fendo.2023.1076253)
Supplement: Supplementary file 2 [file Table_2.pdf]

Intervention	Details
Improving knowledge and awareness:	Video message by a diabetic doctor explaining
Intervention mode	:ABCDs of diabetes: What is diabetes, what are its symptoms/complications. Who is more at risk of developing complications?
	:Healthy HbA1c or fasting blood sugar level, blood pressure or cholesterol levels/modes of treatment
:Video message (5 min) in simple Nepali language	:Why it is important to get blood sugar levels checked regularly by a doctor.
	:Uncontrolled diabetes can affect different vital organs (Show pictures of eye, foot, kidney affected by diabetes)
	:Diabetes, and stress/anxiety are linked: controlling one condition can improve the other
	Video message by a traditional/faith healer explaining that
	:Diabetes is a disease that can be improved only by the treatment or advice given by the doctor
	Video message by a patient who has controlled diabetes successfully
	:Not missing doctor's appointments for diabetic check-ups, not forgetting to take medicine, adopting a healthy lifestyle, etc.
Improving dietary habits/meal planning	Video message by a dietician/diabetic doctor explaining
Intervention mode	:Eating healthy diet is as important as taking medicine.
:Video message (3.5 min) in simple Nepali language	:Appropriate portion size and plate size (9" to 7" plate). Show picture of 'Use your hands to measure out portions
	:Healthy diabetic diet and dietary restrictions for diabetic patients
	:Meals should be consumed in small portions but eat frequently with the 2 hours interval. Eat less white rice.
	:Reduce the amount of fat and ghee in the meal
	:Eat plenty of fruits, vegetables, and pulses along with less quantity of meat (mutton).
	:Eat local foods like oat, buckwheat (fapar), pumpkin seeds, soyabean, dried 'sinki', colacasia (masaura), fenugreek, millet.
:Demonstration by a dietician	:Make a kitchen garden of local fruits/vegetables like methi sag, flax, pumpkin seed, gauva, lemon, and herbs (tulsi, mint)
	:Eat sweet desserts like gulab jamun, peda, rasmalai, jeri, etc. less In religious or cultural events
	:Diabetic diet is not a special diet; instead, it is a well-balanced and healthy diet.
	:Eat less goat meat, reduce intake of ghee, eat in small portions but frequently
Improving physical activity	Video message by a physical activity instructor explaining
	:That physical activity is not just structured exercise and sport or going to gym only.
Intervention mode	:Walking with friends/family members, avoiding long-time sitting are simple exercises that can be done to help diabetes
:Video message (2 min) in simple Nepali language	:Social /family support is important to promote physical exercise
	:Need to engage in physical activity as a habit including yoga. Physical activity does not worsen diabetes
	:Need to stand up at least 2 hours/day for those working in an office; seating needs to be regularly broken up with standing
	:When you travel by bus, get off the bus a few stops before so that you will walk the remaining distance
Improving behaviour through goal setting	Video message by a doctor explaining
Intervention mode	:How goal setting is important to improve healthy behavior for diabetic self-care
:Video message (1 min) in simple Nepali language	:Examples of specific, measurable, achievable, time-bound goal settings appropriate to local culture
Improving diabetic retinopathy screening	Video message by an eye doctor explaining
Intervention mode	:How late visit to eye doctor can increase chances of diabetes-related blindness-show picture of field loss in diabetic retinopathy
:Video message (1.5 min) in simple Nepali language	:Those with longer diabetic duration/family history of diabetes are more likely to develop diabetic retinopathy
	:Diabetes is a silent threat to vision. Patients can have subtle changes in the power of glasses. This should not be ignored

Supplementary Table 2. Details of the diabetic education video program (https://aru.ac.uk/vision-and-eye-research-institute/our-research/diabetic-retinopathy/arudrapp)
