# Supplementary material for: Individual patient-centered target-driven intervention to improve clinical outcomes of diabetes, health literacy, and self-care practices in Nepal: A randomized controlled trial
Source: Front Endocrinol (Lausanne). 2023 Jan 19;14:1076253. doi: 10.3389/fendo.2023.1076253 (PMC9893775; doi:10.3389/fendo.2023.1076253)
Supplement: Supplementary file 3 [file Table_3.pdf]

frontiers

A. Physical activity plan- Time walked/run daily (in minutes)							
Week	Day 1	Day 2	Day 3	Day 4	Day 5	Day 6	Day 7
1							
2							
3							
4							
5							
6							
7							
8							
9							
10							
11							
12							
Targets:							
0 - 4 weeks: 30 minutes walking/running every day for 5 days a week							
4 - 8 weeks: 45 minutes of walking/ running every day for 5 days a week							
8 - 12 weeks: 60 minutes of walking/running every day for 5 days a week							

B. Diet plan		
Timing	Menu/diet	Amount(gm/Kg/Bowl/Cup)
Breakfast		
Lunch		
Snacks		
Mid-noon		
Mid-evening		
Dinner		
Diet targets:		
Reduce the amount of salt, white rice, sweets, tea with sugar by half after six weeks		
Increase the portion of fruits you eat by double after six weeks		
Reduce the portion of purified butter you eat by half after six weeks		
Reduce the portion of fast food you eat by half after six weeks		
Double the amount of leafy vegetables you eat like spinach, and broccoli by six weeks		

Supplementary Table 3. Target-driven physical activity plan (A) and diet plan (B).
